# Multi-Dimensional Performance Evaluation and Basalt Fiber Strengthening Effect of Secondary Hot In-Place Recycled Asphalt Mixtures

**DOI:** 10.3390/ma19102075

**Published:** 2026-05-15

**Authors:** Binhao Su, Jian Hu, Aihong Kang, Yang Zhang

**Affiliations:** College of Civil Science and Engineering, Yangzhou University, Yangzhou 225127, China; mx120230611@stu.yzu.edu.cn (B.S.); mz120241075@stu.yzu.edu.cn (J.H.); zhangyang03130@163.com (Y.Z.)

**Keywords:** asphalt mixture, secondary aging, hot in-place recycling, toughening basalt fiber, pavement performance

## Abstract

To address the rapid performance deterioration and secondary maintenance challenges of highway asphalt pavements that have undergone a first-round hot in-place recycling, this study investigates the feasibility of secondary recycling. Using the Yangzhou section of the G40 Expressway (Class II mild aging) and the Lianyungang section of the G30 Expressway (Class VI severe aging) as engineering backgrounds, three recycling schemes were designed and evaluated: Scheme A (100% RAP control), Scheme B (RAP with rejuvenator and virgin aggregate), and Scheme C (Scheme B reinforced with toughening basalt fibers). A comprehensive multi-dimensional testing protocol—including dynamic stability, semi-circular bending (SCB), low-temperature beam stripping, and Hamburg wheel-tracking—was employed to systematically evaluate the pavement performance of the second-time hot in-place recycled asphalt mixtures. The results indicate that while secondary recycled mixtures (Schemes A and B) maintain acceptable high-temperature stability, their intermediate-to-low temperature cracking resistance serves as the critical bottleneck, failing to meet standard specifications. In contrast, compared with Scheme A (100% RAP control), Scheme C (with basalt fibers) increased the flexibility index by 646.2–946.7%, the low-temperature fracture energy by 96.7–261.0%, and the Hamburg wheel-tracking stripping point by 48.1–62.2%, effectively mitigating the brittle fatigue common in aged recycled binders. According to the Jiangsu Expressway Maintenance Design Guidelines, the incorporation of basalt fibers elevated the comprehensive performance grade of the mixture from below Grade C to Grade A. This research provides a robust scientific basis and a “digital filter” for the large-scale engineering application of sustainable secondary recycling technology in heavy-traffic environments.

## 1. Introduction

As the construction of China’s expressway network approaches completion, pavement maintenance has gradually superseded new construction as the primary focus of road management [1]. By the end of 2025, the total mileage of expressways in Jiangsu Province reached 5560 km, of which asphalt pavements exceeding their designed service life of 15 years accounted for more than 60%, and expressways with a service age of 10 years or above represented as high as 80% of the total network mileage [2,3]. The continuous increase in the overall service age of the road network drives the strategic transformation of Jiangsu’s expressway development objectives from “balancing construction and maintenance” toward “prioritizing maintenance”. Since the introduction of hot in-place recycling equipment in 2002, Jiangsu Province has accumulated nearly 20 years of practical experience in this field, with the cumulative mileage of hot in-place recycling projects exceeding 1500 km [4]. Owing to its outstanding advantages of high efficiency, low carbon footprint, and minimal traffic disruption, this technology has been widely adopted for structural maintenance of expressways across the province. However, according to inspection data from Jiangsu Transportation Holdings, expressways rehabilitated in earlier periods using conventional hot in-place recycling technology have progressively exhibited severe distress, with performance deterioration becoming increasingly pronounced under the coupled effects of long-term traffic loading and environmental exposure [5]. How to implement effective secondary maintenance for such pavements has become a pressing practical challenge in the current field of highway pavement management.

Regarding the feasibility of secondary recycling and the performance of the resulting mixtures, researchers both domestically and internationally have accumulated a relatively rich body of findings [6,7,8,9,10]. Existing studies generally indicate that, although secondary recycled asphalt mixtures are technically feasible, their overall road performance undergoes varying degrees of deterioration compared to first-generation recycled mixtures [6]. Specifically, secondary recycled mixtures perform acceptably in terms of high-temperature stability, yet exhibit pronounced degradation trends in moisture stability, low-temperature cracking resistance, and fatigue resistance [8,9]. Further mechanistic investigations reveal that, following secondary long-term aging, the recycled binder develops improved rutting resistance due to hardening, while its fatigue resistance and resistance to low-temperature cracking are significantly weakened [10].

To address the aforementioned performance deterioration, researchers have conducted systematic investigations at the material design level [11]. By incorporating rejuvenators and virgin asphalt, the conventional components of secondary recycled asphalt can be substantially restored to the level of first-generation recycled material [12]. Building on this foundation, appropriately increasing the rejuvenator dosage and raising the proportion of virgin material to 38% enables the structural stability, moisture stability, and high-temperature stability of secondary hot in-place recycled mixtures to be comprehensively restored, thereby extending pavement service life by four to five years [4]. Meanwhile, key process parameters governing secondary aging behavior have also attracted research attention; the mechanistic role of RAP preheating temperature on the secondary aging behavior of aged asphalt binder [13,14], as well as the influence of RAP content on the secondary aging characteristics of warm-mix recycled asphalt mixtures [15], have both been systematically investigated.

In addition, the incorporation of fiber-based additives has been shown to improve the cracking resistance of recycled asphalt mixtures. Nevertheless, the toughening and strengthening effects of basalt fiber on secondary hot in-place recycled mixtures remain insufficiently studied.

Despite this body of work, several critical gaps remain. First, a systematic multi-dimensional performance evaluation of secondary hot in-place recycled mixtures—covering high-temperature stability, intermediate- and low-temperature cracking resistance, and moisture stability—is still lacking. Notably, comparative studies based on actual pavement sections with distinctly different RAP aging degrees are absent, even though aging severity fundamentally governs the baseline performance and the potential for property recovery. Second, and more importantly, the intermediate-to-low temperature cracking resistance has been consistently identified as the critical bottleneck for secondary recycled mixtures. While adding rejuvenator and virgin materials can partially restore aged binder properties, it cannot adequately overcome the inherent brittleness of severely aged systems. Basalt fiber (BF), with its toughening and crack-bridging mechanisms, offers a promising targeted solution, yet experimental validation in secondary hot in-place recycled mixtures across different aging grades and gradation types remains very limited. The extent to which BF can compensate for the toughness deficiency under realistic multi-dimensional requirements has not been systematically quantified.

In light of the above, this study is conducted based on two actual engineering sections in Jiangsu Province: the Yangzhou section of the Hu-Shan Expressway (G40) (SMA-13 gradation, Grade II light aging) and the Lianyungang section of the Lian-Huo Expressway (G30) (AK-13 gradation, Grade VI severe aging). Three recycling schemes are designed: Scheme A (100% RAP secondary hot in-place recycling), Scheme B (RAP + virgin aggregate + rejuvenator), and Scheme C (RAP + virgin aggregate + rejuvenator + toughening BF). A systematic investigation of the road performance of secondary hot in-place recycled asphalt mixtures is carried out through a comprehensive test program comprising wheel-tracking tests, dynamic creep tests, intermediate-temperature semi-circular bending (SCB) tests, low-temperature beam bending tests, low-temperature SCB tests, immersion Marshall tests, freeze–thaw splitting tests, and Hamburg wheel-tracking tests. The road performance of each recycling scheme is systematically evaluated from the perspectives of high-temperature stability, moisture stability, low-temperature cracking resistance, and intermediate-temperature cracking resistance, with multi-dimensional performance grading conducted in accordance with the Jiangsu Highway Asphalt Pavement Maintenance Design Guide. To fill the identified gaps, this study aims to: (1) systematically evaluate the multi-dimensional performance of secondary recycled mixtures derived from RAP materials of different aging grades and identify the controlling bottleneck; and (2) quantify the toughening effects of BF as a targeted means of overcoming the brittleness limitation of secondary recycled systems. The findings are intended to provide a scientific basis for the large-scale engineering application and comprehensive performance improvement of secondary hot in-place recycling technology.

It should be noted that the quantitative findings of this study are specific to the RAP sources, aging grades, and mixture types investigated. While the two gradation types—SMA-13 (gap-graded, skeleton-dense) and AK-13 (dense-graded, suspended-dense)—represent two common aggregate skeleton configurations on Chinese expressways, the generalizability of the reported numerical improvements to other mixture types, aging conditions, or climatic regions should be verified through site-specific validation.

## 2. Materials and Methods

### 2.1. Raw Materials

#### 2.1.1. Reclaimed Asphalt Pavement (RAP)

The reclaimed asphalt pavement (RAP) materials used in this study are sourced from major maintenance projects on the Yangzhou section of the Hu-Shan Expressway (G40) and the Lianyungang section of the Lian-Huo Expressway (G30), respectively. The G40 section was originally constructed with an SMA-13 upper layer and opened to traffic in December 2012, while the G30 section was originally constructed with an AK-13 upper layer; both pavements used SBS-modified asphalt in their original construction. Both sections underwent their first hot in-place recycling in 2019, and the RAP materials sampled for this study were collected prior to the secondary hot in-place recycling operation carried out in 2025.

**Figure 1:**
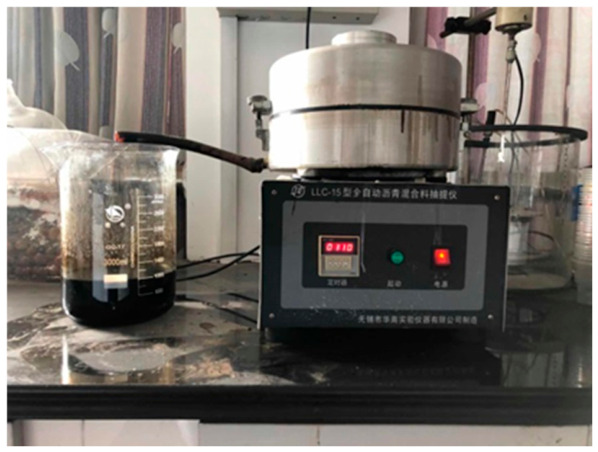
Centrifugal extraction test chart [16].

**Figure 2:**
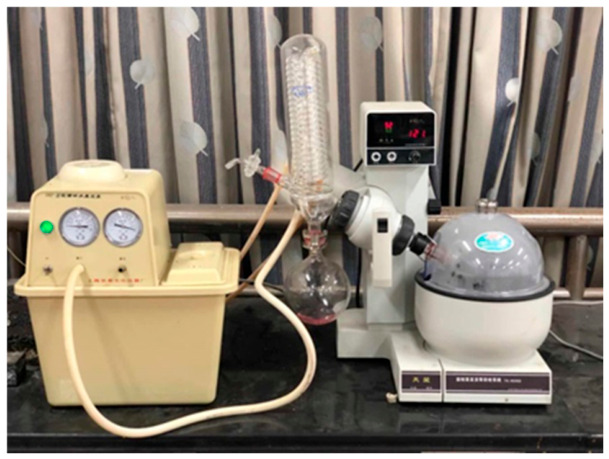
Rotational evaporation test diagram [16].

The aged binder is recovered from the RAP using the centrifugal extraction method combined with rotary evaporation, as illustrated in Figure 1 and Figure 2 [16]. The recovered binder is characterized in terms of penetration, softening point, and ductility in accordance with JTG E20 [17]. The results show that the aged binder from the G40 section exhibits a 25 °C penetration of 35.4 (0.1 mm) and a 135 °C viscosity of 2.06 Pa·s, which corresponds to Grade II light aging according to the aging classification standard for aged SBS-modified asphalt [18]. The aged binder from the G30 section exhibits a 25 °C penetration of 16.4 (0.1 mm) and a 135 °C viscosity of 10.4 Pa·s, corresponding to Grade VI severe aging. The two sections thus exhibit markedly different degrees of RAP aging.

Extraction and sieve analysis tests are performed on the RAP materials from both sections, with results presented in Table 1. The asphalt-to-aggregate ratio is 6.0% for the G40 section and 4.5% for the G30 section. Analysis of the gradation and asphalt content data in Table 1 indicates that the passing rates at all sieve sizes fall within the upper and lower limits of the original design gradation, confirming that the aggregate gradation remains largely intact. This demonstrates that both RAP materials meet the prerequisites for secondary recycling in accordance with the original gradation.

#### 2.1.2. SBS-Modified Asphalt

Virgin asphalt in the form of SBS-modified binder is used, with a penetration of 71 (0.1 mm), softening point of 80 °C, 5 °C ductility of 48 cm, and elastic recovery of 76%. All properties comply with the requirements of the Technical Specifications for Construction of Highway Asphalt Pavements (JTG F40—2004) [19].

#### 2.1.3. Virgin Aggregate and Mineral Filler

All virgin aggregates are basalt aggregates, and the filler consists of limestone mineral powder. Aggregate properties including density and water absorption are tested in accordance with the Test Methods of Aggregate for Highway Engineering, with results presented in Table 2. All technical indicators meet the requirements of the corresponding specifications.

#### 2.1.4. Toughening Basalt Fiber

**Figure 3:**
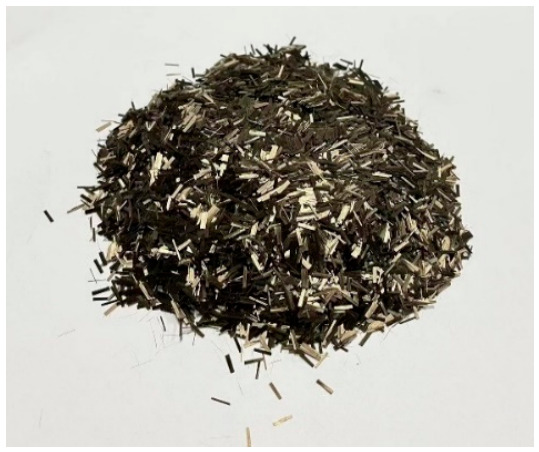
Toughening Basalt Fiber.

The toughening BF adopted in this study is shown in Figure 3, with its primary technical specifications listed in Table 3. The fiber exhibits a breaking strength exceeding 2000 MPa and a heat-resistant breaking strength retention rate of no less than 93%. All indicators satisfy the requirements of Fiber for Asphalt Pavement (JT/T 776) [20].

### 2.2. Mix Design

**Figure 4:**
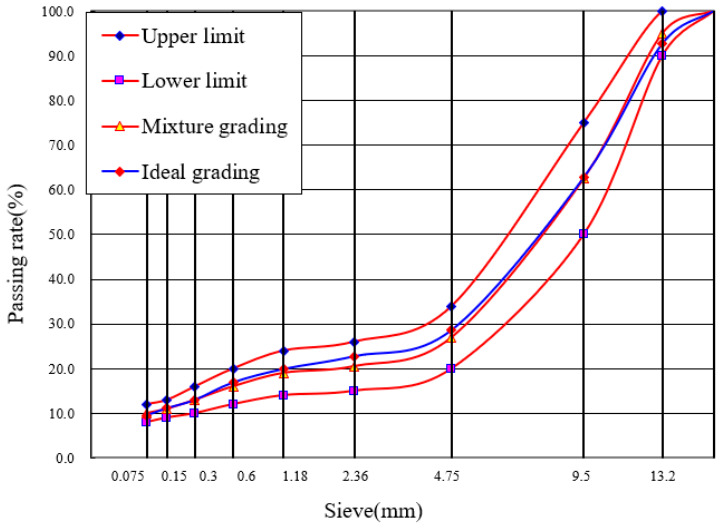
Recycled Synthetic Gradation of SMA-13.

**Figure 5:**
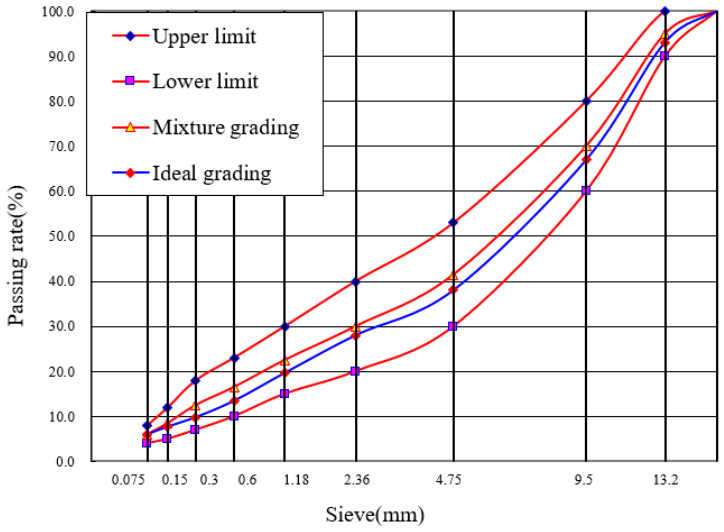
Recycled Synthetic Gradation of AK-13.

Based on the gradation characteristics of the reclaimed asphalt pavement (RAP) materials and the mix design of virgin materials, the recycled composite gradations of the two mixtures were determined. The corresponding mix proportion parameters are summarized in Table 4, while the gradation curves are presented in Figure 4 and Figure 5.

Marshall validation tests were then conducted on specimens prepared according to the above mix design schemes. The results showed that the air voids, Marshall stability, retained stability, freeze–thaw splitting strength ratio, dynamic stability, and low-temperature bending failure strain of the SMA-13 and AK-13 recycled asphalt mixtures all satisfied the specification requirements [24]. The validation results are presented in Table 5.

### 2.3. Recycling Schemes

To systematically evaluate the contribution of each constituent material to mixture performance, three secondary in-place recycling schemes are designed in this study (Table 6). Scheme A serves as the control group consisting entirely of reclaimed material (R) (100% RAP), representing the baseline performance of the original pavement material. Scheme B incorporates virgin aggregate (N) and a rejuvenator (RA) into the reclaimed material, representing a conventional hot recycling approach. Scheme C further introduces toughening basalt fiber (BF) into Scheme B, enabling evaluation of the comprehensive fiber-toughening effect.

Based on the gradation characteristics of the RAP materials and the mix design of the virgin aggregate, the blended target gradations for the two mixture types are determined. All mix design parameters are summarized in Table 4, and the gradation curves are presented in Figure 4 and Figure 5. Marshall verification tests are conducted on specimens prepared from each mix design scheme. The void ratio, Marshall stability, residual stability ratio, freeze–thaw splitting strength ratio, dynamic stability, and low-temperature bending failure strain of both the SMA-13 and AK-13 recycled mixtures all satisfy the specification requirements, with verification results presented in Table 6.

### 2.4. Experimental Methods

All experimental tests were conducted using a minimum of three replicate specimens per mixture type.

#### 2.4.1. High-Temperature Stability Test

(1) Rutting test

The rutting test was conducted in accordance with JTG E20 T0719-2011 [17]. After conditioning in an environment of 60 ± 1 °C for no less than 5 h, the specimens were subjected to a wheel load pressure of 0.7 MPa. Dynamic stability (DS) was adopted as the principal evaluation index.

(2) Dynamic creep test

Referring to NCHRP 9-29 [25], the dynamic creep test was performed using a UTM-25 servo-hydraulic multifunctional material testing system (MTS Systems Corporation, Eden Prairie, MN, USA). The specimens were cylindrical samples with a diameter of 100 mm and a height of 150 mm. The test was carried out at 60 °C under an axial stress of 0.7 MPa, with a loading regime consisting of a 0.1 s haversine load pulse followed by a 0.9 s rest period, until either the cumulative microstrain reached 100,000 or the number of load cycles reached 10,000. The creep rate and flow number (Fn) were used as evaluation indices. Among them, Fn denotes the critical number of load cycles at which the material enters the tertiary creep stage. A higher Fn value indicates a stronger resistance to permanent deformation.

#### 2.4.2. Intermediate-Temperature Cracking Resistance Test

In accordance with AASHTO TP 124-16 [26], the semicircular bending (SCB) test was employed to evaluate the cracking resistance of the mixtures at normal service temperature.

The test was conducted on a UTM-25 testing machine at 15 °C with a loading rate of 50 mm/min (MTS Systems Corporation, Eden Prairie, MN, USA). Fracture energy (Gf) and flexibility index (FI) were taken as the primary evaluation indices.

#### 2.4.3. Low-Temperature Cracking Resistance Test

(1) Beam bending test

According to JTG E20 [17], the beam bending test was carried out at a temperature of −10 °C and a loading rate of 50 mm/min. The flexural tensile strength (RB), maximum flexural tensile strain (εB), and bending stiffness modulus (SB) were used as evaluation indices.

(2) Low-temperature SCB test

With reference to the enterprise standard of Jiangsu Transport Holding Co., Ltd. (Nanjing, China), O/SJKG YH B2-001-2020 [27] Maintenance Design Guidelines for Asphalt Pavement of Jiangsu Expressways, the same specimens as those used in the intermediate-temperature SCB test were adopted. The test was performed on the UTM-25 testing machine at −10 °C with a loading rate of 50 mm/min, and fracture energy ($G_{f}$) was used as the evaluation index.

(1) $$G_{f}=\frac{W_{f}}{A_{lig}}$$

(2) $$W_{f}=\int Pdu$$

(3) $$A_{lig}=(r-a)t$$

where *G_f_* is the fracture energy, J/m^2^; *W_f_* is the fracture work, J; *A_lig_* is the ligament area, mm^2^; *r* − *a* is the ligament length, mm; *t* is the specimen thickness, mm.

#### 2.4.4. Moisture Stability Test

(1) Immersion Marshall test and freeze–thaw splitting test

The immersion Marshall test and the freeze–thaw splitting test were conducted in accordance with JTG E20 [17]. The moisture stability of the mixtures was characterized by the retained Marshall stability after immersion (MS_0_) and the tensile strength ratio obtained from the freeze–thaw splitting test.

(4) $$MS_{0}=\frac{MS_{1}}{MS}\times 100$$

where MS_0_ is the retained Marshall stability of the specimen, %; MS is the Marshall stability, kN; MS_1_ is the stability of the specimen after 48 h of water immersion, kN.

(5) $$TSR=\frac{{\bar{R}}_{T2}}{{\bar{R}}_{T1}}$$

where TSR is the tensile strength ratio of the freeze–thaw splitting test, %; R_T2_ is the average splitting tensile strength of the second group of valid specimens after the freeze–thaw cycle, MPa; and R_T1_ is the average splitting tensile strength of the first group of valid specimens without the freeze–thaw cycle, MPa.

(2) Hamburg wheel-tracking test

The Hamburg wheel-tracking test was conducted in accordance with AASHTO T324 [28]. Gyratory-compacted specimens with a diameter of 150 mm and a height of 60 mm were cut and mounted in molds for testing. Under a 60 °C water bath condition, wheel tracking was performed at a wheel load pressure of 0.7 MPa and a loading frequency of 52 passes/min until either 20,000 loading cycles were reached or the rut depth reached 20 mm. The principal evaluation indices included the maximum rut depth, creep slope, stripping slope, and stripping inflection point, which collectively reflect the stripping resistance and moisture damage resistance of the mixture under coupled water–heat conditions [29].

## 3. Result

### 3.1. High-Temperature Stability of Secondary Recycled Asphalt Mixtures

#### 3.1.1. Wheel-Tracking Test Results and Analysis

**Figure 6:**
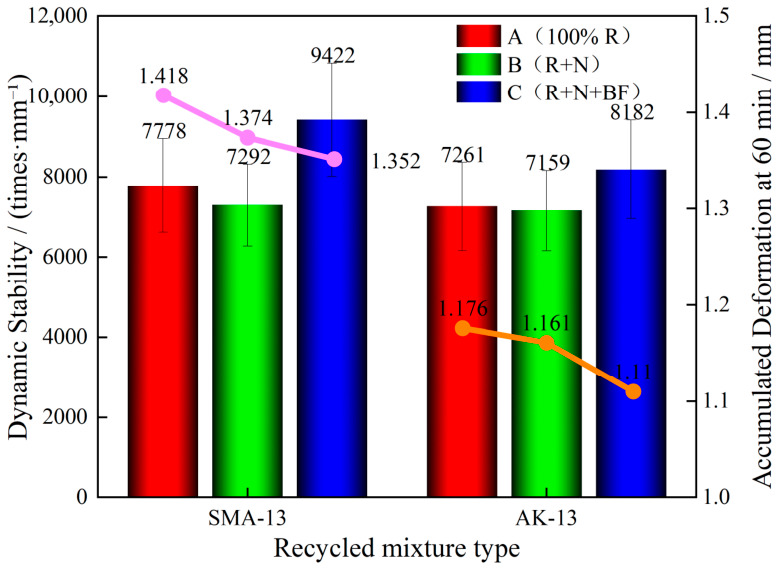
Rutting Test Results.

As shown in Figure 6, for both SMA-13 and AK-13 gradation mixtures, the dynamic stability of the 100% RAP mixtures exceeded 7000 cycles/mm, reaching 7778 cycles/mm for SMA-13 and 7261 cycles/mm for AK-13, both far surpassing the specification threshold of ≥3000 cycles/mm and demonstrating excellent rutting resistance. The dynamic stability of the Scheme B mixtures decreased slightly, falling to 7292 cycles/mm for SMA-13 and 7159 cycles/mm for AK-13. This reduction was attributed to the incorporation of rejuvenator and virgin aggregate, which improved the flowability of the originally aged and hardened binder, thereby reducing its viscosity.

Compared with the Scheme B secondary recycled mixtures, the Scheme C mixtures exhibited notably higher dynamic stability. The dynamic stability of the SMA-13 and AK-13 secondary recycled mixtures increased by 29.2% and 14.3%, reaching 9422 cycles/mm and 8182 cycles/mm, respectively. This improvement was attributed to the ability of toughening basalt fiber (BF) to adsorb free asphalt within the mixture, thereby enhancing the adhesion effect and improving high-temperature stability [30]. In addition, basalt fiber forms a three-dimensional network structure with the aggregate and binder within the mixture, generating frictional resistance in conjunction with its reinforcing function, which impedes binder flow between aggregates and suppresses inter-aggregate relative sliding, thereby enhancing the rutting resistance of the asphalt mixture [31].

For both gradation types, the dynamic stability of the SMA-13 recycled mixtures was slightly higher than that of the AK-13 mixtures. This was because AK-13 adopts a suspended dense structure, in which the coarse aggregates remain in a suspended state and lack a skeletal framework to provide structural support. Under high-temperature service conditions, this configuration is prone to excessive deformation and rutting. In contrast, SMA-13 employs a skeleton-dense structure, wherein the coarse aggregates form a compact interlocking skeleton that suppresses high-temperature deformation and effectively slows rutting development [32]. In summary, the secondary hot in-place recycled asphalt mixtures demonstrate excellent high-temperature rutting resistance, far exceeding the specification requirement of ≥3000 cycles/mm.

#### 3.1.2. Dynamic Creep Test Results and Analysis

The Secondary Stage Model listed in Table 7 represents the linear regression equation fitted to the steady-state creep stage of the cumulative microstrain vs. load cycle curve, from which the creep rate (slope) and flow number (Fn) are derived. The model was established by least-squares linear regression of the data points within the identified secondary stage region of each creep curve.

**Figure 7:**
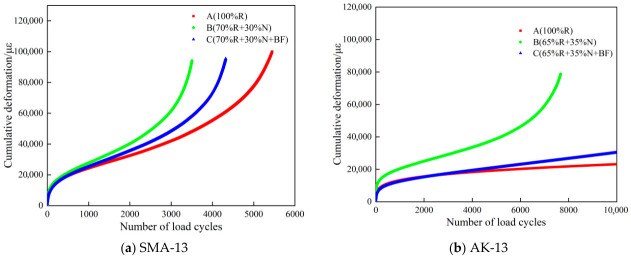
Curve of Cumulative Strain versus Number of Load Applications.

As revealed by Figure 7 and Table 7, both gradation types exhibit similar trends: the 100% RAP recycled mixtures show relatively low creep rates, whereas the Scheme B mixtures exhibit higher creep rates, indicating that the original RAP material possesses superior shear resistance. Notably, the AK-13 mixture with 100% RAP exhibited the most stable creep behavior, with a creep rate of only 0.9, and no creep failure was observed. This was because the aged binder in the RAP underwent a certain degree of hardening and stiffening, which increased the overall shear strength of the recycled mixture. Mixtures with higher shear strength are less susceptible to softening and deformation under high-temperature conditions [33,34].

Compared with the Scheme B secondary recycled mixtures, the Scheme C mixtures showed significantly reduced creep rates and markedly increased flow numbers. Taking the SMA-13 mixture as an example, the adoption of Scheme C reduced the creep rate by 12.6% (from 12.7 to 11.1) and increased the flow number from 2350 to 3076, demonstrating that the incorporation of basalt fiber effectively improved the high-temperature deformation resistance of the recycled mixture. Basalt fiber adsorbs asphalt binder to reduce free binder flow, thereby preserving the internal structural integrity and stability of the mixture, enhancing cohesion, and making the mixture more resistant to deformation and failure under high-temperature conditions [31,35]. Similar trends were observed for the AK-13 secondary recycled mixtures. Comparing the results of the wheel-tracking and dynamic creep tests, both sets of results exhibit consistent trends and mutually corroborate each other.

The secondary hot in-place recycled asphalt mixtures prepared under both aging backgrounds demonstrate satisfactory high-temperature deformation resistance, with high-temperature stability meeting engineering requirements. While different recycling schemes exert a certain influence on high-temperature performance, the differences manifest only as quantitative variations in performance level rather than qualitative transitions from compliance to non-compliance, indicating that high-temperature stability does not represent the controlling performance bottleneck for secondary hot in-place recycled mixtures. Scheme C achieves a moderate improvement in high-temperature stability while maintaining its performance advantage, suggesting that the introduction of toughening basalt fiber does not compromise the load-bearing capacity of the mixture’s aggregate skeleton structure and contributes to enhanced structural stability under high-temperature loading.

It should be noted that the larger cumulative strains observed for SMA-13 relative to AK-13 in the Dynamic Creep Test are predominantly a consequence of the substantial difference in binder aging state: the AK-13 RAP is severely aged (Grade VI), yielding a much stiffer binder and hence lower creep strains, whereas the SMA-13 RAP is only lightly aged (Grade II). This observation underscores the complementary nature of the Dynamic Creep Test and the wheel-tracking test in characterising high-temperature performance.

### 3.2. Intermediate-Temperature Performance of Secondary Recycled Asphalt Mixtures

**Figure 8:**
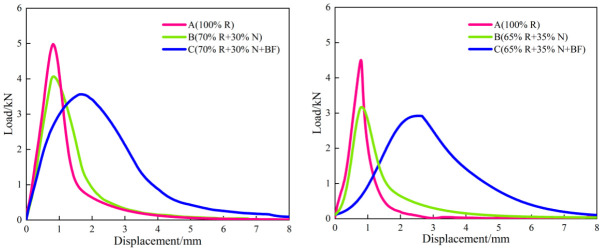
Load–Displacement Curve of SCB Test.

**Figure 9:**
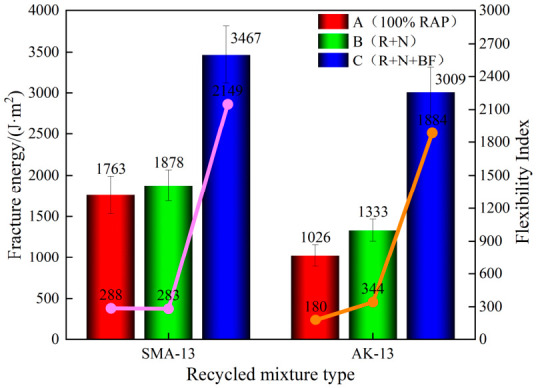
SCB Test Results.

The load–displacement curves of the SCB test of the recycled asphalt mixtures are presented in Figure 8, and the SCB test results are shown in Figure 9.

At a test temperature of 15 °C, the secondary hot in-place recycled asphalt mixtures underwent viscoelastic deformation under loading before ultimately fracturing, exhibiting a characteristic response of moderate deformation followed by rapid failure and cracking. The fracture energy Gf of the 100% RAP mixtures was only 1763 J/m^2^ (SMA-13) and 1026 J/m^2^ (AK-13), with flexibility indices (FI) of only 288 and 180, respectively, and a peak displacement of approximately 0.80 mm. These results indicate that, following one cycle of hot in-place recycling, the RAP material had lost a portion of its elasticity and flexibility, rendering it more susceptible to cracking and fracture under loading.

Compared with the 100% RAP mixtures, the Scheme B mixtures showed a certain degree of improvement in cracking resistance, though the enhancement was limited: the fracture energy Gf of the SMA-13 and AK-13 mixtures increased by 6.5% (1878 J/m^2^) and 29.9% (1333 J/m^2^), respectively, while the flexibility index FI improved by 33.0% (383) and 91.1% (344), respectively. After recycling with Scheme C, the cracking resistance of the mixtures was significantly enhanced. Compared with the 100% RAP mixtures, the fracture energy G_f of the two gradation types increased by 96.7% (SMA-13: 3467 J/m^2^) and 193.3% (AK-13: 3009 J/m^2^), respectively, and the flexibility index FI improved by 646.2% (SMA-13: 2149) and 946.7% (AK-13: 1884), respectively, with peak displacement rising from approximately 0.80 mm to approximately 2.0 mm. These results demonstrate the pronounced strengthening and toughening effect of the toughening basalt fiber, which forms bridging fibers throughout the interior of the mixture, generating closure stresses across the two fracture surfaces and thereby constraining crack initiation and propagation.

The contrasting improvements between Scheme B and Scheme C reveal a fundamental difference in their toughening mechanisms. The addition of rejuvenator and virgin asphalt (Scheme B) primarily restores the chemical composition of the aged binder, but this chemical restoration alone cannot adequately repair the compromised aggregate–binder interface that has been embrittled by long-term aging. Consequently, cracks, once initiated, still propagate readily, and the improvement in FI remains limited. In contrast, BF (Scheme C) physically bridges microcracks, transfers stress across crack faces, and dissipates fracture energy through fiber pull-out and debonding [35,36]. This mechanical intervention directly addresses the crack propagation phase, explaining why the FI of Scheme C mixtures improved by 646–947%, whereas Scheme B achieved only 33–91% improvement. For context, previous studies on BF-reinforced primary recycled mixtures have reported FI improvements in the range of 55–75% [33]; the substantially larger enhancement observed in this study underscores the critical role of BF as a targeted toughening agent for severely aged secondary systems.

### 3.3. Low-Temperature Performance of Secondary Recycled Asphalt Mixtures

#### 3.3.1. Low-Temperature Beam Bending Test Results and Analysis

This test was performed in accordance with JTG E20 (T0715) [17].

**Figure 10:**
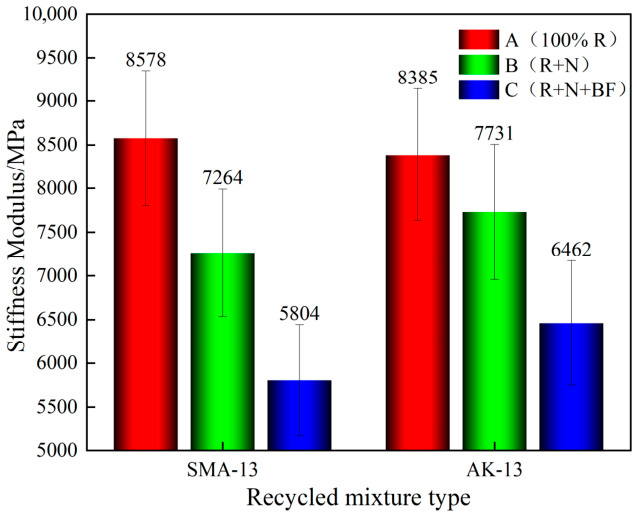
Intermediate-Temperature SCB Test Results.

**Figure 11:**
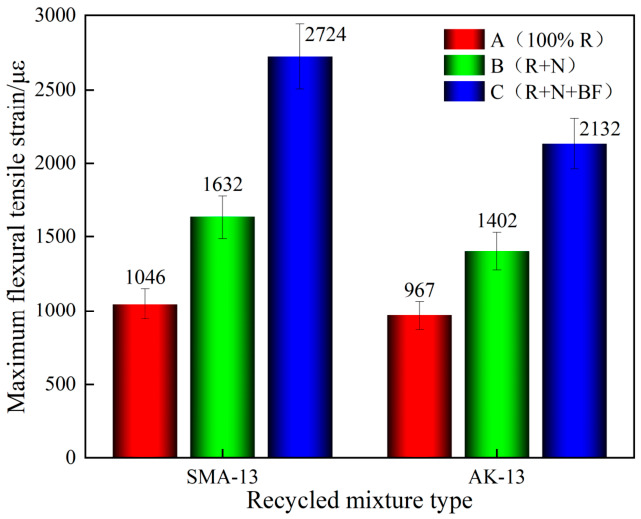
Comparison of Flexural Stiffness Modulus.

As shown in Figure 10 and Figure 11, the maximum flexural tensile strain of the 100% RAP recycled mixtures for both SMA-13 and AK-13 gradations was only approximately 1000 με (SMA-13: 1046 με; AK-13: 967 με), far below the design requirement of ≥2000 με. This indicates that low-temperature cracking resistance had deteriorated substantially following one cycle of hot in-place recycling. After secondary recycling with Scheme B, the maximum flexural tensile strain recovered to some extent, but the improvement was limited and the results still did not satisfy the specification requirements. Upon incorporation of toughening basalt fiber (Scheme C), the maximum flexural tensile strain increased markedly. Compared with the 100% RAP mixtures, the maximum flexural tensile strain of the SMA-13 mixtures recycled under Schemes B and C increased by 56.0% and 160.4%, respectively; for the AK-13 mixtures, the corresponding increases were 45.0% and 120.5%, reaching 2724 με (SMA-13) and 2132 με (AK-13), both satisfying the design requirement of ≥2000 με.

The marked increase in maximum flexural tensile strain achieved by Scheme C is attributed to the crack-arresting function of BF. At −10 °C, the aged binder becomes extremely brittle and loses most of its deformation capacity. Under these conditions, the embedded BF—with a tensile strength exceeding 2000 MPa and an elongation at break of approximately 2.7%—acts as a bridging element across microcracks. As cracks propagate through the brittle matrix, the fibers absorb energy through elastic elongation and eventual pull-out, thereby redistributing stress away from the crack tip and delaying catastrophic failure [36,37]. This mechanism is particularly critical for the severely aged G30 section (AK-13, Grade VI), where the binder has lost nearly all its inherent ductility. It is noteworthy that despite the substantial improvement from Scheme C, the maximum flexural tensile strain of the AK-13 recycled mixture only marginally satisfies the ≥2000 με requirement, indicating that low-temperature cracking resistance remains the most difficult property to restore in severely aged secondary systems. This finding is consistent with the observation that BF-reinforced composites exhibit superior energy absorption under low-temperature conditions due to the thermal stability and high modulus of basalt fibers [28].

#### 3.3.2. Low-Temperature SCB Test Results and Analysis

**Figure 12:**
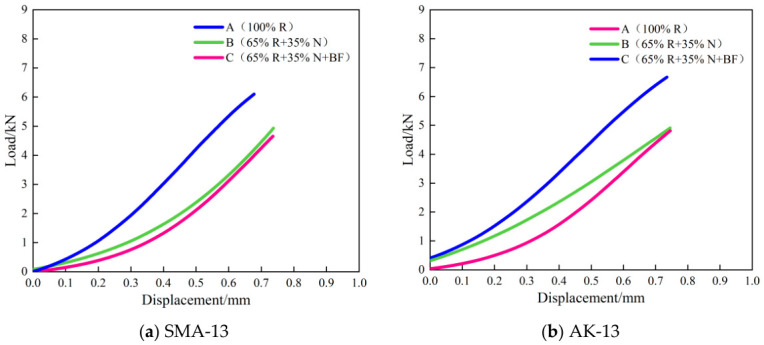
Load–Displacement Curve of the SCB Test under Low-Temperature Conditions.

**Figure 13:**
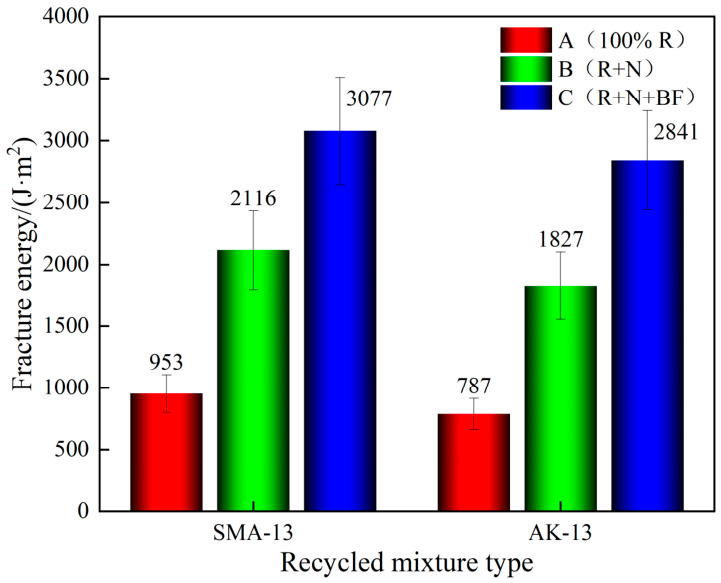
Fracture Energy Comparison.

As shown in Figure 12, at a test temperature of −10 °C, the secondary hot in-place recycled asphalt mixtures exhibited brittle fracture under loading. As shown in Figure 13, the fracture energy of the 100% RAP mixtures was significantly low, at 953 J/m^2^ for SMA-13 and 787 J/m^2^ for AK-13. Compared with the 100% RAP mixtures, the fracture energy of the SMA-13 and AK-13 Scheme B mixtures increased by 122.0% (2116 J/m^2^) and 132.1% (1827 J/m^2^), respectively; for the Scheme C mixtures, the corresponding increases were 222.9% (3077 J/m^2^) and 261.0% (2841 J/m^2^), respectively.

The trends observed in this test are consistent with those from the low-temperature beam bending tests, and the two sets of results mutually corroborate each other. The improvement in low-temperature fracture energy achieved by Scheme C substantially exceeds that of Scheme B. This is attributed to the fact that the toughening basalt fiber, upon incorporation, forms an integrated load-bearing system with the asphalt mixture, jointly resisting external loading. The high tensile strength of the basalt fiber enables the mixture to absorb greater energy under stress, thereby further enhancing low-temperature cracking resistance [37].

The low-temperature test results reveal marked differences in the capacity for cracking resistance recovery among mixtures derived from pavement materials with different degrees of aging. Mixtures from severely aged pavement sections are more prone to embrittlement under low-temperature conditions, and the greater the degree of raw material aging, the more difficult it is to restore low-temperature cracking resistance after recycling. Therefore, the performance evaluation of secondary hot in-place recycled mixtures should not be confined to assessing post-recycling acceptability alone, but should also address the degree of performance recovery under different aging backgrounds. Scheme C significantly improves both low-temperature failure strain and fracture energy, demonstrating that basalt fiber can function as a bridging and energy-dissipating element during low-temperature crack initiation and propagation, effectively compensating for the toughness deficiency inherent in aged recycled systems.

### 3.4. Moisture Stability of Secondary Recycled Asphalt Mixtures

#### 3.4.1. Immersion Marshall Test and Freeze–Thaw Splitting Test

**Figure 14:**
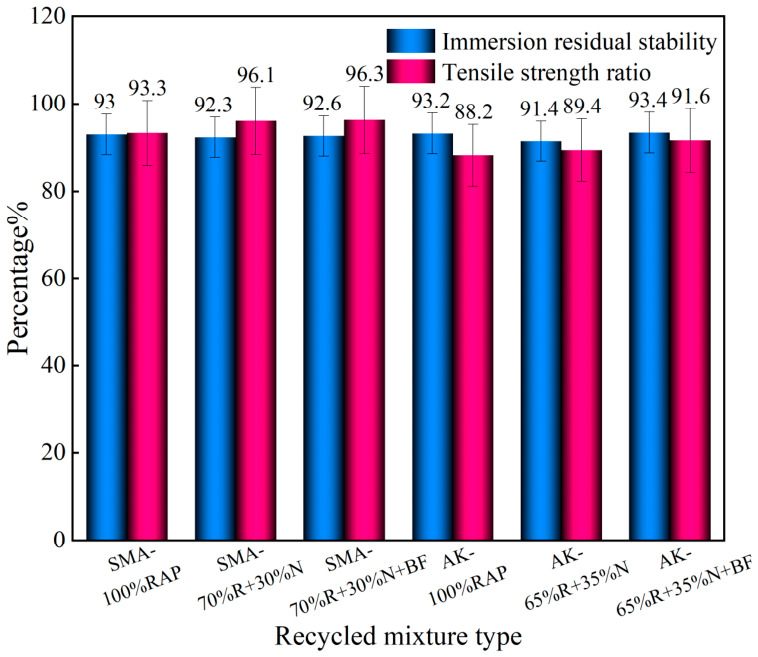
Water Stability Test Results.

As shown in Figure 14, for both SMA-13 and AK-13 gradations, the freeze–thaw splitting strength ratios of the Scheme B and Scheme C mixtures were slightly higher than those of the 100% RAP mixtures. The immersion residual Marshall stability of all recycled mixtures met the specification requirement of ≥85% (minimum value: 91.4%), and the freeze–thaw splitting strength ratio satisfied the requirement of ≥80% (minimum value: 88.2%), indicating that the moisture stability of the recycled mixtures is satisfactory and has not undergone notable deterioration. As revealed by the comparative analysis in Table 8, the 100% RAP mixtures exhibited the most severe maximum rut depth (SMA-13: 11.02 mm; AK-13: 15.72 mm), along with the highest creep rates and stripping rates and the lowest stripping points (SMA-13: 9928 cycles; AK-13: 6868 cycles), indicating that the 100% RAP recycled mixtures performed poorly under coupled moisture-thermal conditions, with significantly reduced resistance to moisture-induced stripping.

#### 3.4.2. Hamburg Wheel-Tracking Test Results

In Table 8, the maximum rut depth is the final rut depth at test termination; the creep slope and stripping slope are fitted to the deformation-dominated and moisture-damage-dominated phases, respectively; and the stripping inflection point is their intersection.

**Figure 15:**
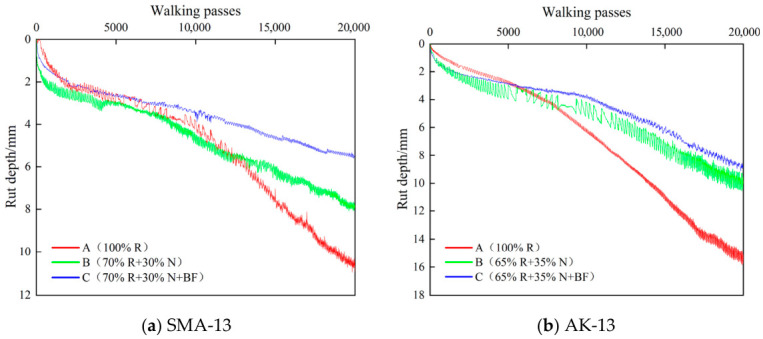
Hamburg Wheel Tracking Curve.

As shown in Figure 15, after recycling with Scheme B, the stripping point of the SMA-13 mixture increased from 9928 to 11,211 cycles, and that of the AK-13 mixture increased from 6868 to 10,172 cycles, representing improvements of 12.9% and 31.9%, respectively. After recycling with Scheme C, the stripping point of the SMA-13 mixture increased from 9928 to 13,097 cycles, and that of the AK-13 mixture increased from 6868 to 11,142 cycles, representing improvements of 48.1% and 62.2%, respectively, demonstrating that toughening basalt fiber effectively enhances the moisture stripping resistance of asphalt mixtures. Compared with the immersion Marshall test and freeze–thaw splitting test, the Hamburg wheel-tracking test exhibits greater sensitivity and applicability for evaluating the moisture stability of secondary hot in-place recycled mixtures under coupled moisture-thermal conditions. The addition of BF increases the compactness and cohesion of the mixture, further improving its resistance to moisture-induced stripping.

The conventional moisture stability indicators of the mixtures under all recycling schemes performed well, confirming that secondary hot in-place recycled mixtures possess a solid foundation of resistance to moisture damage. The Hamburg wheel-tracking test results reveal that, under coupled moisture-thermal conditions, meaningful differences in damage resistance persist among different recycling schemes, suggesting that conventional single-indicator approaches have limited capacity to discriminate durability differences among materials. Scheme C demonstrates superior performance in terms of both stripping point and deformation resistance, indicating that the beneficial effect of basalt fiber extends beyond cracking resistance to encompass enhanced binder film stability, improved aggregate–binder interfacial synergy, and increased overall resistance to moisture damage, reflecting a comprehensive performance enhancement for secondary recycled mixtures.

### 3.5. Comprehensive Multi-Dimensional Performance Grading Evaluation

The preceding sections individually examined the performance differences of secondary hot in-place recycled mixtures from the perspectives of high-temperature deformation, intermediate-temperature cracking, low-temperature brittleness, and moisture damage. However, conclusions drawn from individual indicators are inherently partial and insufficient to comprehensively reflect the engineering suitability of materials prepared under different recycling schemes. In a secondary recycling system, the various performance dimensions do not necessarily change in synchrony: satisfactory high-temperature performance does not preclude service risk, and inadequate intermediate-to-low-temperature cracking resistance may still constitute the critical factor constraining long-term pavement performance. It is therefore necessary to introduce a multi-dimensional performance grading evaluation framework beyond individual test results, as summarized in Table 9 and Table 10, enabling unified assessment of the comprehensive road performance of mixtures under different aging backgrounds and recycling schemes, with the aim of identifying performance bottlenecks and quantifying the actual contribution of basalt fiber to overall performance improvement.

As shown in Table 11, in terms of high-temperature stability, all six recycled asphalt mixtures satisfied the Grade B requirement (i.e., flow number F_n > 2000). The SMA-13 mixture recycled under Scheme C further satisfied the Grade A requirement (i.e., F_n ≥ 3000). The AK-13 mixtures recycled under Schemes A and B did not exhibit creep failure, demonstrating outstanding high-temperature deformation resistance.

Regarding intermediate-temperature durability, the SMA-13 and AK-13 mixtures recycled under Schemes A and B both failed to meet the Grade C requirement, indicating that RAP aging leads to a loss of elasticity and flexibility, rendering the mixtures more susceptible to cracking and fracture. After recycling with Scheme C, the flexibility index FI of both gradation types satisfied the Grade A requirement, demonstrating a substantial improvement in intermediate-temperature durability.

In terms of low-temperature cracking resistance, the fracture energy G_f of the 100% RAP secondary recycled mixtures for both gradation types was below 1000 J/m^2^, failing to meet the Grade C requirement. The markedly low fracture energy values indicate that low-temperature cracking resistance had deteriorated significantly following one cycle of hot in-place recycling. After recycling with Scheme C, the fracture energy of both SMA-13 and AK-13 mixtures satisfied the Grade A requirement, confirming that low-temperature cracking resistance was effectively restored.

Overall, the secondary hot in-place recycled mixtures perform well in terms of high-temperature stability and conventional moisture stability, providing a viable foundation for engineering application. However, in terms of intermediate-temperature and low-temperature cracking resistance—particularly under high-RAP-content conditions without fiber incorporation—performance deterioration is pronounced, and insufficient cracking resistance represents the core constraint on broader application. Scheme B yields partial improvements in certain performance dimensions but provides limited remediation of the cracking resistance bottleneck. Scheme C, through the introduction of toughening basalt fiber, achieves significant enhancement of intermediate-to-low-temperature cracking indicators for both gradation types, elevating the comprehensive performance grade from below Grade C to Grade A. This demonstrates that basalt fiber functions as a targeted reinforcing agent against the brittleness deficiency of secondary recycled mixtures, thereby substantially improving their overall road performance and engineering suitability.

## 4. Conclusions

This study systematically evaluates the high-temperature stability, intermediate-temperature cracking resistance, low-temperature cracking resistance, and moisture stability of secondary hot in-place recycled asphalt mixtures prepared from pavement materials with different degrees of aging under three recycling schemes. The main conclusions are as follows:

(1) Under the three recycling schemes, the secondary hot in-place recycled asphalt mixtures all exhibited excellent high-temperature stability and satisfactory moisture stability, with all indicators meeting the specification requirements, indicating their basic feasibility for engineering application. However, the 100% RAP mixture (Scheme A) showed significantly low intermediate-temperature flexibility and low-temperature bending strain. Insufficient intermediate-to-low temperature cracking resistance is the critical performance bottleneck limiting its wider application. The addition of virgin aggregate and rejuvenator (Scheme B) provided only limited improvement and could not fundamentally resolve the toughness deterioration. The effectiveness of the BF-reinforcement approach may vary with the aging severity of the RAP source and the climatic conditions of the project site, and site-specific validation is recommended before large-scale application.

(2) With the combined use of virgin aggregate, rejuvenator, and basalt fiber (Scheme C), the high-temperature performance was further optimized: dynamic stability increased by 14.3–29.2%, and creep rate decreased by 12.6–57.8%. The improvement in intermediate-to-low temperature cracking resistance was even more pronounced: Intermediate-temperature flexibility index increased by 646.2–946.7%; Low-temperature flexural strain increased by 120.5–160.4%; Low-temperature fracture energy increased by 96.7–261.0%; Hamburg wheel-tracking stripping point extended by 48.1–62.2%. The reinforcing mechanism of basalt fiber is primarily manifested as bridging, restraining, and energy dissipation during crack propagation, effectively enhancing the overall toughness of the recycled mixtures.

(3) According to the multi-dimensional performance grading system of the Jiangsu Expressway Asphalt Pavement Maintenance Design Guide, the overall performance grades of both SMA-13 and AK-13 mixtures under Scheme C were elevated from below Grade C to Grade A, satisfying the service requirements for heavy and above traffic. Toughening basalt fiber provides targeted remediation of the cracking resistance bottleneck of secondary recycled mixtures and significantly improves their comprehensive road performance, demonstrating clear engineering application potential.

(4) In summary, secondary hot in-place recycled asphalt mixtures demonstrate satisfactory baseline performance in terms of high-temperature stability and moisture stability, while insufficient intermediate-to-low-temperature cracking resistance constitutes the core constraint on their engineering application. Toughening basalt fiber effectively addresses this critical performance deficiency and significantly elevates the comprehensive performance grade of the mixtures, representing a strengthening and toughening pathway with clear engineering application potential. The findings of this study provide a scientific basis and technical reference for the large-scale promotion and application of secondary hot in-place recycling technology.

Future research should focus on validating the long-term field performance of secondary recycled mixtures reinforced with basalt fiber under actual traffic and environmental conditions, exploring the synergistic effects of different fiber types and dosages, and conducting life-cycle cost and environmental benefit analyses to further support the sustainable application of this technology.

## Figures and Tables

**Table 1:** Extraction Test Results of RAP Materials.

Section	Asphalt–Aggregate Ratio (%)	Sieve Size (mm) Passing Rate (%)									
		16.0	13.2	9.5	4.75	2.36	1.18	0.6	0.3	0.15	0.075
G40	6.0	100.0	92.0	60.0	28.8	22.6	19.0	15.8	12.1	10.8	9.4
G30	4.50	100.0	94.2	68.4	40.3	31.1	21.0	14.4	10.7	8.6	6.6

**Table 2:** Density and Water Absorption Test Results of Aggregates and Mineral Filler.

Aggregate Size	Apparent Relative Density (g/cm^3^)	Bulk Relative Density (g/cm^3^)	Water Absorption (%)
10–15 mm	2.952	2.749	1.26
5–10 mm	2.944	2.744	1.47
3–5 mm	2.930	2.777	1.59
0–3 mm	2.863	-	-
Mineral Filler	2.712	-	-

**Table 3:** Key Technical Properties of Toughening Basalt Fiber (HIRBF-G13).

Test Item	HIRBF-G13	Technical Requirement	Test Method
Diameter (μm)	17	±10%	GB/T 7690.5 [21]
Length (mm)	6	±10%	JT/T 776.1 [20]
Tensile Strength (MPa)	2460	≥2000	GB/T 20310 [22]
Elongation at Break (%)	2.7	≥2.1	GB/T 20310 [22]
Elastic Modulus (GPa)	85.8	≥80	GB/T 20310 [22]
Heat Resistance Strength Retention (%)	93.6	≥85	JT/T 776.1 [20]
Acidity Coefficient	6.0	≥5.0	GB/T 1549 [23]

**Table 4:** Mix Design Parameters of Secondary Recycled Asphalt Mixtures.

Gradation Type	New Material (%)	RAP (%)	Rejuvenator Content (%)	Optimum Asphalt Content (%)	Basalt Fiber (BF) Content (%)	Theoretical Maximum Relative Density (g/cm^3^)
SMA-13	30	70	3.0	5.9	0.25	2.617
AK-13	35	65	4.0	4.85	0.30	2.600

**Table 5:** Marshall Verification Results of Recycled Asphalt Mixtures.

Item	SMA-13 Test Result	SMA-13 Requirement	AK-13 Test Result	AK-13 Requirement	Test Method JTG 3410-2025 [24]
Air Voids (%)	4	3–4.5	5.4	4.0–5.5	T0705
Stability (kN)	10.2	≥6.0	15.4	≥8.0	T0709
Marshall Residual Stability (%)	92.6	≥85	93.4	≥85	T0709
Freeze–Thaw Splitting Strength Ratio (%)	96.3	≥80	91.6	≥80	T0729
Dynamic Stability (passes·mm^−1^)	8764	≥3000	6347	≥3000	T0719
Low-Temperature Bending Failure Strain (με)	2136	≥2000	2132	≥2000	T0715

**Table 6:** Definition of Recycling Methods for Secondary Hot In-Place Recycled Asphalt Mixtures.

Recycling Method	SMA-13 Mixture Composition	AK-13 Mixture Composition
A	100% R	100% R
B	70% R + 30% N + 3% RA	65% R + 35% N + 4% RA
C	70% R + 30% N + 3% RA + BF	65% R + 35% N + 4% RA + BF

**Table 7:** Dynamic Creep Test Results.

Mixture Type		Secondary Stage Model (y = ax + b)	R^2^ (-)	Creep Rate (με/Cycle)	Flow Number, Fn (Cycles)
SMA-13	A (100% RAP)	y = 9.6x + 13,951	0.991	9.6	3836
	B (70% R + 30% N)	y = 12.7x + 14,935	0.999	12.7	2350
	C (70% R + 30% N + BF)	y = 11.1x + 13,970	0.999	11.1	3076
AK-13	A (100% RAP)	y = 0.9x + 14,405	0.980	0.9	—
	B (65% R + 35% N)	y = 4.5x + 15,648	0.983	4.5	5169
	C (65% R + 35% N + BF)	y = 1.9x + 11,474	0.998	1.9	—

**Table 8:** Hamburg Wheel Tracking Test Results.

Mixture Type		Composition	Maximum Rut Depth (mm)	Creep Slope	Stripping Inflection Point (Passes)
SMA-13	A (100% RAP)	11.02	2.5 × 10^−4^	7.0 × 10^−4^	9928
	B (70% R + 30% N)	7.99	2.3 × 10^−4^	3.4 × 10^−4^	11,211
	C (70% R + 30% N + BF)	5.53	1.6 × 10^−4^	2.1 × 10^−4^	13,097
AK-13	A (100% RAP)	15.72	4.0 × 10^−4^	9.1 × 10^−4^	6868
	B (65% R + 35% N)	10.33	2.8 × 10^−4^	5.5 × 10^−4^	10,172
	C (65% R + 35% N + BF)	8.75	2.0 × 10^−4^	4.8 × 10^−4^	11,142

**Table 9:** Test Methods and Evaluation Indices for Performance.

Performance	Test Method	Evaluation Index
High-temperature stability	Standard Dynamic Creep Test	Flow Number (Fn)
Intermediate-temperature durability	15 °C Semi-Circular Bending (SCB) Test	Flexibility Index (FI)
Low-temperature cracking resistance	−10 °C Semi-Circular Bending (SCB) Test	Fracture Energy (Gf)

**Table 10:** Indoor Performance Requirements for Hot In-Place Recycled Asphalt Mixtures.

Grade	Flow Number (Fn)	Intermediate-Temperature Flexibility Index (FI)	Fracture Energy Gf (J/m^2^)	Applicable Conditions	Reference
A	≥3000	>1000	≥2800	\	[38]
B	[2000, 3000)	[700, 1000)	[2000, 2800)	Heavy traffic and above	
C	[1500, 2000)	[500, 700)	[1000, 2000)	Medium to light traffic	

**Table 11:** Indoor Performance Grade Classification of Mixture.

Mixture Type	Fn	Fn Grade	FI	FI Grade	Gf (J·m^−2^)	Gf Grade	Overall Grade
SMA-13-A	3836	A	288	<C	953	<C	<C
SMA-13-B	2350	B	383	<C	2116	B	<C
SMA-13-C	3076	A	2149	A	3077	A	A
AK-13-A	—	A	180	<C	787	<C	<C
AK-13-B	5169	A	344	<C	1827	C	<C
AK-13-C	—	A	2149	A	2841	A	A

## Data Availability

The original contributions presented in this study are included in the article. Further inquiries can be directed to the corresponding author.

## Nomenclature

The following notations were used in this paper:

BF	Basalt fiber
RAP	Reclaimed Asphalt Pavement
RA	Rejuvenating Agent
SCB	Semi-circular bending
SMA-13	Stone–mastic–asphalt with 13.2 mm nominal maximum aggregate size
AK-13	Anti-rutting asphalt concrete with 13.2 mm nominal maximum aggregate size
